# Use of Sentiment Analysis for Capturing Patient Experience From Free-Text Comments Posted Online

**DOI:** 10.2196/jmir.2721

**Published:** 2013-11-01

**Authors:** Felix Greaves, Daniel Ramirez-Cano, Christopher Millett, Ara Darzi, Liam Donaldson

**Affiliations:** ^1^Department of Primary Care and Public HealthImperial College LondonLondonUnited Kingdom; ^2^Centre for Health PolicyImperial College LondonLondonUnited Kingdom

**Keywords:** Internet, patient experience, quality, machine learning

## Abstract

**Background:**

There are large amounts of unstructured, free-text information about quality of health care available on the Internet in blogs, social networks, and on physician rating websites that are not captured in a systematic way. New analytical techniques, such as sentiment analysis, may allow us to understand and use this information more effectively to improve the quality of health care.

**Objective:**

We attempted to use machine learning to understand patients’ unstructured comments about their care. We used sentiment analysis techniques to categorize online free-text comments by patients as either positive or negative descriptions of their health care. We tried to automatically predict whether a patient would recommend a hospital, whether the hospital was clean, and whether they were treated with dignity from their free-text description, compared to the patient’s own quantitative rating of their care.

**Methods:**

We applied machine learning techniques to all 6412 online comments about hospitals on the English National Health Service website in 2010 using Weka data-mining software. We also compared the results obtained from sentiment analysis with the paper-based national inpatient survey results at the hospital level using Spearman rank correlation for all 161 acute adult hospital trusts in England.

**Results:**

There was 81%, 84%, and 89% agreement between quantitative ratings of care and those derived from free-text comments using sentiment analysis for cleanliness, being treated with dignity, and overall recommendation of hospital respectively (kappa scores: .40–.74, *P*<.001 for all). We observed mild to moderate associations between our machine learning predictions and responses to the large patient survey for the three categories examined (Spearman rho 0.37-0.51, *P*<.001 for all).

**Conclusions:**

The prediction accuracy that we have achieved using this machine learning process suggests that we are able to predict, from free-text, a reasonably accurate assessment of patients’ opinion about different performance aspects of a hospital and that these machine learning predictions are associated with results of more conventional surveys.

## Introduction

Understanding patients’ experience of health care is central to the process of providing care and is a fundamental pillar of health care quality [1,2]. Traditional measures of patient experience include surveys, and more recently, structured patient reported outcome measures. Such approaches ask specific and limited questions, are conducted infrequently, and are often expensive to administer. Today’s patients have begun to report their health care experience on the Internet in blogs, social networks, wikis, and on health care rating websites [3,4]. However, as this is largely unstructured, nonstandardized free-text information, it is not captured in a systematic way. This represents a missed opportunity for understanding patients’ experience in an increasingly “connected” world. Survey data in the United States suggest that 85% of adults use the Internet [5], 25% have read someone else’s experience about health on a website or blog, and 11% have consulted online reviews of hospitals or other medical facilities [6].

Outside health care, natural language processing of large datasets, including sentiment analysis and opinion mining, has been critical to understanding consumer attributes and behaviors, for example, in election forecasting [7,8]. Sentiment analysis enables the content of natural language—the words we write and speak—to be examined for positive and negative opinions and emotion [9]. If applicable to health care, these analytical methods could permit interpretation of textual information about patient experience on a huge scale. This information, because of its prose nature, has avoided the analytical spotlight of conventional quantitative analysis. Alemi and colleagues have proposed the use of sentiment analysis of comments as real-time patient surveys [10]. They have shown that patient comments about specific doctors could be attributed with reasonable accuracy to positive and negative sentiment. They further suggest that capture of sentiment analysis should be compared to traditional methods of assessing patient experience.

The Information Strategy for the National Health Service (NHS) in England states that sentiment analysis of data could be a novel source of information [11] that would be valuable for patients in facilitating choice of hospitals. We tested this assertion and furthered the work undertaken by Alemi and colleagues, by analyzing a large number of free-text comments on the main NHS website (NHS Choices). This website allows patients to describe their treatment experiences at all hospitals in England (and all other NHS-provided services). It is well used, with around a million hits a day. Over a 2-year period, each hospital had a mean average of 69 reviews [12]. These reviews include both free-text descriptions of general experience and Likert-scale ratings of particular aspects of care. This presents the opportunity for a natural experiment to assess the accuracy of sentiment analysis techniques (applied to the free-text comments) against the patients’ own quantitative ratings. The NHS also measures patient experience via a national survey of hospital inpatients. If sentiment analysis techniques are to be considered as useful tools for assessing care quality, it is important to see whether there is an association with traditional measures of patient experience. We therefore compare our sentiment analysis findings to the national patient survey, at the hospital level.

## Methods

### Machine Learning From Patient Comments

We applied data processing techniques to all the online free-text comments about hospitals on the NHS Choices website in 2010. Our purpose was to test whether we could automatically predict patients’ views on a number of topics from their free-text responses. A machine learning classification approach was chosen in which an algorithm “learns” to classify comments into categories from a given set of examples, using open-source Weka data mining software. This software has been extensively used in previous research and provides accurate classification results, including in health care [13-15]. To test the accuracy of the prediction, we compared our results to quantitative ratings provided by the same individual patients on a Likert scale. Free-text comments were examined in response to the questions: “What I liked”, “What could have been improved”, and “Any other comments”. A prediction was then made about whether the patient would recommend the hospital or not, whether the hospital was clean or dirty, and whether they were treated with dignity and respect. The algorithm was trained using all comments and ratings about hospitals left on the NHS Choices website from 2008, 2009, and 2011 (13,802 in total) as a learning set. Data from 2010 were used to test the predicting accuracy of the process (6412 comments) because comparable patient experience survey data were available for that year. The validation set represents 31.7% of the total sample available. We performed a single round of cross validation. All comments left on the NHS Choices website were provided directly by the Department of Health in England but have subsequently been made publicly available [16].

### Technical Aspects of the Machine Learning Approach

The machine learning approach had two components: (1) pre-processing, in which data from patient comments are split into manageable units to build a representation of the data [17], and (2) classification, in which an algorithm decides which category each comment falls into. A consistent set of methodologies was applied in our machine learning process, including a “bag-of-words” approach, “prior polarity”, and “information gain”.

In the “bag-of-words” approach, the total body of words analyzed (known as the corpora) is represented as a simplified, unordered collection of words [18]. For this analysis, unigrams (single elements or words) and bigrams (two adjacent elements in a string of tokens, in this case, a 2-word phrase) were used as the basic units of analysis. We extracted 5695 n-grams in total. Higher n-grams (longer phrases) could have been used, but the constraints were computer power and processing time. We also included our own classification of certain words in the machine learning approach, known as “prior polarity”. The 1000 most common single words, and the 1000 most common 2-word phrases were extracted from the complete set of comments in the corpora. Two researchers independently rated the sentiment of each as positive, negative, either, or neutral separately for each of the three domains under consideration: (1) overall recommendation, (2) cleanliness, and (3) dignity. Where disagreements occurred, the sentiment was discussed and resolved between the 2 researchers. Kappa statistics for overall ratings were .76 for 1 word and .71 for 2-word phrases. For rating of dignity, they were .71 for 1 word and .70 for 2 words. For rating of cleanliness, they were .52 for 1 word and .48 for 2 words. For all of these calculations, *P*<.001.

A technique called “information gain” was used to reduce the size of the bag-of-words by identifying those words with the lowest certainty of belonging to a given class, and then removing them—this is an approach to feature selection [19]. This improved the computation time and also demonstrates the words with highest predictive accuracy.

A number of different technical approaches can be taken to classification in machine learning. We applied four different methods, to see which gave the quickest and most accurate results: (1) naïve Bayes multinomials (NBM) [20], (2) decision trees [21], (3) bagging [22], and (4) support vector machines [23]. Decision trees and bagging were carried out with REPTree in the Weka package. Support vector machines used an RBF Kernel. The accuracy of the prediction was compared with the patient’s own quantitative rating by calculating, for each method, the accuracy (the percentage of correctly predicted observations from the total number of observations), the *F* measure (the harmonic mean of precision and recall), the Receiver Operating Characteristic (ROC), and the time taken to complete the task were calculated. To reduce computing processing time of the classification, we limited the words in the learning process to the top 10,000 words by frequency. All text was converted to lower case, and we removed all punctuation. Typographical errors and misspellings were not corrected.

### Testing Prediction Accuracy

To obtain a score to predict sentiment analysis against, patient ratings left on the NHS Choices website on a Likert scale were converted into simple categories, either positive or negative about cleanliness and dignity, to simplify the prediction task. The website presents patients with five options to rate the cleanliness of a hospital: “exceptionally clean”, “very clean”, “clean”, “not very clean”, “dirty”, and “does not apply”. In this analysis, the first three options were grouped into a “clean” class and the “not very clean” and “dirty” into a “dirty” class. The website also asks patients to rate whether they were treated with dignity and respect by the hospital staff, with the options being “all of the time”, “most of the time”, “some of the time”, “rarely”, and “not at all”. Again, the first three options were grouped, in this case into a “more dignity” class and the “rarely” and “not at all” into a “less dignity” class. Finally, the NHS Choices website asks all patients whether they would recommend the hospital or not.

### Comparing Sentiment Analysis With the National Inpatient Survey

Having calculated the accuracy of our prediction algorithm, the results of the sentiment analysis were then compared with the national inpatient survey results for 2010. This is an annual national survey of randomly selected patients admitted to NHS hospitals in England, similar to the HCAHPS survey in the United States. The 2010 survey covered all 161 acute hospitals with adult services in England, involving 60,000 respondents nationally (response rate 50%). Patients were contacted via post between September 2010 and January 2011 if they had received overnight care in hospital in 2010 [24]. This survey includes both general and specific questions. In this study, we used only areas similar to the specific themes predicted from the NHS Choices data. The questions used were “In your opinion, how clean was the hospital room or ward that you were in?” (marked on a 4-point scale of “very” to “not at all”); “Overall, did you feel you were treated with respect and dignity while you were in the hospital?” (marked on a 3-point scale of “very” to “not at all”); and “Overall, how would you rate the care you received?” (marked on a 5-point scale from excellent to poor). The sentiment analysis ranking was compared with the patient survey ranking for each question at the hospital level, applying Spearman rank correlation, using Stata SE11 statistical software. We compared all 161 adult acute trusts in England.

## Results

There was agreement between the patients’ own quantitative rating of whether they would recommend their hospital and our prediction from sentiment analysis between 80.8% and 88.6% of the time (expressed as accuracy; Table 1), depending on the classification method used. Similarly, sentiment analysis agreed with whether the patient was treated with dignity and respect between 83.7% and 84.5% of the time, and agreed with whether the hospital was clean or not between 81.2% and 89.2% of the time. Table 2 shows the 10 words or 2-word phrases with the highest predictive accuracy.

NBM, bagging, and decision tree approaches to classification all produced similar measures of association, but the NBM algorithm, a first-order probabilistic model that uses word frequency information, performed the calculation faster (less than 0.2 seconds compared to hundreds of seconds for the other analyzed approaches). Of note, all algorithms tended to be worse at predicting cleanliness and SVM in particular. This may represent the limited language around cleanliness compared to the other opinions examined, or the more skewed results, with higher number of negative ratings.

**Figure 1:**
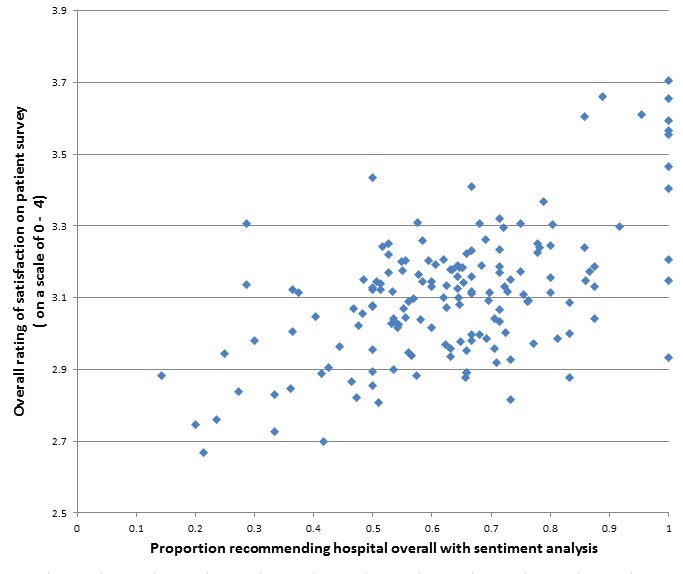
Comparison of the proportion recommending a hospital using sentiment analysis and traditional paper-based survey measures.

On this basis, we choose to use NBM results for further comparison of our results with patient survey data. The relationship between the predictions of the NBM approach and the real ratings was reflected as Kappa statistics for interrater reliability of between .40 and .74 (*P*<.001 for all). We found significant, weak to moderate associations between machine learning predictions using NBM and quantitative responses from the national inpatient survey for the three categories examined: cleanliness, dignity, and overall recommendation (Spearman correlation coefficients between 0.37 and 0.51, *P*<.001 for all) (see Table 3). Rank correlations for overall recommendations of hospitals are displayed in Figure 1.

**Table 1:** Accuracy of different approaches to machine learning.

Question		Overall rating	Cleanliness	Dignity and respect
**Naïve Bayes multinomial**				
	ROC	0.94	0.88	0.91
	*F* measure	0.89	0.84	0.85
	Accuracy (%)	88.6	81.2	83.7
	Time (s)	0.11	0.05	0.06
**Decision trees**				
	ROC	0.84	0.76	0.79
	*F* measure	0.81	0.86	0.8
	Accuracy (%)	80.8	88.4	83
	Time (s)	552	206	332
**Bagging**				
	ROC	0.89	0.83	0.87
	*F* measure	0.82	0.87	0.85
	Accuracy (%)	82.5	89.2	84.5
	Time (s)	4871	2018	3164
**Support vector machine**				
	ROC	0.79	0.53	0.6
	*F* measure	0.84	0.84	0.8
	Accuracy (%)	84.6	88.5	84.1
	Time (s)	612	305	520

**Table 2:** The 10 one or two word phrases with the highest predictive accuracy for each topic.

Overall	Cleanliness	Dignity
told	dirty	rude
thank you	floor	told
left	left	left
rude	the floor	thank you
excellent	thank you	friendly
the staff	filthy	excellent
hours	bed	rude and
asked	patients	asked
was told	friendly	the staff
friendly	hours	staff

**Table 3:** Comparison of patient survey responses and machine learning prediction of comments at hospital trust level.

Patient survey question	Machine learning prediction	Spearman correlation coefficient	Probability
In your opinion, how clean was the hospital room or ward that you were in?	Machine learning prediction of comments about standard of cleanliness	0.37	*P*<.001
Overall, did you feel you were treated with respect and dignity while you were in the hospital?	Machine learning prediction of comments about whether the patient was treated with dignity and respect	0.51	*P*<.001
Overall, how would you rate the care you received?	Machine learning prediction of comments about whether the patient would recommend	0.46	*P*<.001

## Discussion

### Principal Findings

Our results reinforce earlier findings that sentiment analysis of patients’ online comments is possible with a reasonable degree of accuracy [10] and that it is possible to identify salient aspects of reviews [25]. We have also shown that unstructured comments in free text, if processed appropriately, are associated with patient experience results from paper-based surveys conducted annually across all hospitals in England. This is in keeping with previous work that has demonstrated that there is a significant association between structured online ratings of care left on health care review websites and conventional surveys of satisfaction [12]. These results suggest a potential mechanism to make use of the large amounts of text on the Internet in which people describe their care, and that further exploration of the information contained within the free-text comments may be an important avenue for understanding patient experience, providing an additional source of information to complement traditional survey methods.

### Strengths and Limitations

Sentiment analysis via a machine learning approach is only as good as the learning set that is used to inform it. By taking advantage of a complete national rating system over several years, we have been able to use many more ratings in this learning set than in other studies. Indeed, our learning set was more than 10 times larger than earlier work [9]. Moreover, in applications of sentiment analysis to health care data, researchers have had to train the system themselves by reviewing comments and ascribing characteristics to them, to allow the algorithm to learn. We used a large dataset that permitted us to directly compare free-text comments and quantitative ratings posted by the same patients, thus eliminating potential biases of reviewer assignment of comments. Similarly, the consistency of questions across the NHS inpatient survey and the questions asked on the NHS Choices website about health services allows for a direct comparison of patient comments and surveys that we believe has not previously been reported.

Online comments left without solicitation on a website are likely to have a natural selection bias towards examples of both good and bad care. It is likely that these online reviews are contributed more by those in particular demographic groups including younger and more affluent people [26]. Further, there are aspects of patients’ comments that are very hard for sentiment analysis to process. Irony, sarcasm, and humor, frequently adopted by English speakers when talking about their care, cannot be easily detected using this process. The use of prior polarity improved the results and mitigated some colloquial phrasing, but there were difficulties understanding those that depend on context. For example, phrases that cropped up repeatedly, such as “stank of urine” or “like an angel”, could be easily characterized as negative or positive. The meaning of other frequently used phrases, however, was hard to establish without an understanding of their context. The best example of this was the phrase a “cup of tea”. It was referred to in many different comments in these data, but without knowing the context, is it impossible to allocate it a direct sentiment. ‘”They didn’t even offer me a cup of tea” is very different to “The nurse even made me a cup of tea”. Our current algorithm could not yet make use of references to cups of tea or similar phrases that would be clear and obvious looked at by eye on a case-by-case basis. Future attempts to improve a natural language processing ability for patient experience would have to develop the capacity to accurately interpret this level of context-specific and idiomatic content. We appreciate that in this early exploratory work, we are not using the most state-of-the-art machine learning algorithms seen in other industries [27], or approaches to classification selection [28], but hope that further work might be able to adopt this.

### Further Research Questions

Further research is needed to improve the performance of sentiment analysis tools, extending the process to other forms of free-text information on the Internet and exploring the relationships between views expressed by patients online and clinical health care quality. For example, several technical components might be added to improve the process, including the consideration of higher number n-grams (longer phrases) and refining contextual polarity (understanding what a word or phrase means given its context in a sentence). It would also be useful to compare the relatively simple techniques used in this analysis against other platforms and tools used for the sentiment analysis and opinion mining process, for example WordNet Affect [29] and SentiWordNet [30,31].

### Policy Implications

Large amounts of data about the use of services are collected in digital form. An important strand of this is consumer opinion and experience. Today, many people express their views and share their experience of goods and services via the Internet and social media. Such data, converted to information, are essential in improving services, facilitating consumer choice and, in some sectors, exploring public accountability and value in the use of taxpayers’ money.

By its nature, the information is highly personalized, idiosyncratic, and idiomatic. However, if it is to be useful, it must be analyzed in ways that are not solely reliant on someone reading individual contributions (although this is valuable to consumers) nor on pre-structured responses necessary to allow aggregation. A solution to the challenge of “big data” is to find automated methods for analyzing unstructured narrative commentary, which is a potentially rich source of learning. In this respect, health care is no different to many other industries although it has perhaps been slower than other sectors to recognize the importance of it.

As our confidence in techniques of data mining and sentiment analysis grow, information of this sort could be routinely collected, processed, and interpreted by health care providers and regulators to monitor performance. Moreover, information could be taken from a number of different text sources online, such as blogs and social media. If this information could be harvested from these locations and then processed into timely and relevant data, it could be a valuable tool for quality improvement. We have previously suggested that as the usage of rating websites, social networks, and microblogs increases [3,4], this free-text information represents a growing and largely untapped source of data that could be considered a “cloud of patient experience” [32]. Others, including Alemi, have discussed similar ideas, with Cambria and colleagues describing a notion of “crowd validation” of a health service [10,33]. This has the potential to provide up-to-date information about patient experience at lower cost than the traditional survey route. It might also allow the views of younger, more tech savvy groups—who are often poor responders to paper-based surveys—to be sampled. Eventually, there might even be the potential to develop a close to real-time early warning system for poor clinical care, if large enough amounts of data can be collected and prediction accuracy can be reliably reproduced. However, caution should be taken before placing too much faith in a quantitative approach, as the qualitative analysis of information of this sort is known to provide useful insights [34]. Qualitative and quantitative approaches should be seen as complementary.

### Conclusions

This work demonstrates that sentiment analysis of patients’ comments about their experience of health care is possible and that this novel approach is associated with patient experience measured by traditional methods such as surveys. This work adds to a growing body of literature opening up a new understanding of the patients’ point of view of care from their postings online—on social networks, blogs, and rating websites. Although at an early and experimental stage, it presents future possibilities to understand health care system performance in close to real time. Bates and colleagues have described the confluence of patient-centered care and social media as a “perfect storm” that is likely to be of major value to the public and to health care organizations [35]. These early findings hint at how that might occur.

## Abbreviations

HCAHPS: Hospital Consumer Assessment of Healthcare Providers and Systems
NBM: Naïve Bayes Multinomial
NHS: National Health Service
NHSC: NHS Choices
ROC: Receiver Operating Characteristic
SVM: Support Vector Machine

## References

[1] Institute of Medicine (2001). Crossing the Quality Chasm: A New Health System for the 21st Century.

[2] Darzi A (2008). High quality care for all: NHS Next Stage Review final report.

[3] Gao GG, McCullough JS, Agarwal R, Jha AK (2012). A changing landscape of physician quality reporting: analysis of patients' online ratings of their physicians over a 5-year period. J Med Internet Res.

[4] Greaves F, Millett C (2012). Consistently increasing numbers of online ratings of healthcare in England. J Med Internet Res.

[5] (2012). Demographics of internet users.

[6] Fox S (2011). The Social Life of Health Information.

[7] Tumasjan A, Sprenger T, Sandner P, Welp I (2010). Predicting elections with Twitter: what 140 characters reveal about political sentiment.

[8] Holzinger A, Yildirim P, Geier M, Simonic KM, Pasi G (2013). Quality-based knowledge discovery from medical text on the Web example of computational methods in Web intelligence. Advanced Techniques in Web Intelligence - Quality-based Information Retrieval Studies in computational intelligence, Lecture Notes in Artificial Intelligence, LNAI.

[9] Pang B, Lee L (2008). Opinion mining and sentiment analysis found. Trends Inf Retr.

[10] Alemi F, Torii M, Clementz L, Aron DC (2012). Feasibility of real-time satisfaction surveys through automated analysis of patients' unstructured comments and sentiments. Qual Manag Health Care.

[11] (2012). The Power of Information: Putting all of us in control of the health and care information we need.

[12] Greaves F, Pape UJ, King D, Darzi A, Majeed A, Wachter RM, Millett C (2012). Associations between Internet-based patient ratings and conventional surveys of patient experience in the English NHS: an observational study. BMJ Qual Saf.

[13] Frank E, Hall M, Trigg L, Holmes G, Witten IH (2004). Data mining in bioinformatics using Weka. Bioinformatics.

[14] Sigurdardottir AK, Jonsdottir H, Benediktsson R (2007). Outcomes of educational interventions in type 2 diabetes: WEKA data-mining analysis. Patient Educ Couns.

[15] Ivanciuc O (2008). Weka machine learning for predicting the phospholipidosis inducing potential. Curr Top Med Chem.

[16] (2013). NHS Choices.

[17] Petz G, Karpowicz M, Fürschuß H, Auinger A, Winkler S, Schaller S, Holzinger A (2012). On text preprocessing for opinion mining outside of laboratory environments. Active Media Technology, Lecture Notes in Computer Science.

[18] Zhang Y, Jin R, Zhou ZH (2010). Understanding bag-of-words model: a statistical framework. Int J Mach Learn & Cybe.

[19] Yang Y, Pedersen JO (1997). A comparative study on feature selection in text categorization.

[20] McCallum A, Nigam K (1998). A comparison of event models for naive bayes text classification.

[21] Quinlan JR (1986). Induction of decision trees. Mach Learn.

[22] Breiman L (1996). Bagging predictors. Mach Learn.

[23] Cortes C, Vapnik V (1995). Support-vector networks. Mach Learn.

[24] (2012). Acute Trusts: Adult Inpatients Survey (2010) Economic and Social Data Service.

[25] Brody S, Elhadad N (2010). Detecting salient aspects in online reviews of health providers. AMIA Annu Symp Proc.

[26] Greaves F, Pape UJ, Lee H, Smith DM, Darzi A, Majeed A, Millett C (2012). Patients' ratings of family physician practices on the internet: usage and associations with conventional measures of quality in the English National Health Service. J Med Internet Res.

[27] Liu B (2012). Sentiment analysis and opinion mining. Synthesis Lectures on Human Language Technologies.

[28] Demsar J (2006). Statistical comparisons of classifiers over multiple data sets. Journal of Machine Learning Research.

[29] Strapparava C, Valitutti A (2004). WordNet affect: an affective extension of WordNet.

[30] Baccianella S, Esuli A, Sebastiani F (2010). SentiWordNet 3.0. An enhanced lexical resource for sentiment analysis and opinion mining.

[31] Esuli A, Sebastiani F (2006). Sentiwordnet: a publicly available lexical resource for opinion mining.

[32] Greaves F, Ramirez-Cano D, Millett C, Darzi A, Donaldson L (2013). Harnessing the cloud of patient experience: using social media to detect poor quality healthcare. BMJ Qual Saf.

[33] Cambria E, Hussain A, Havasi C (2010). Towards the crowd validation of the British National Health Service.

[34] Lagu T, Goff SL, Hannon NS, Shatz A, Lindenauer PK (2013). A mixed-methods analysis of patient reviews of hospital care in England: implications for public reporting of health care quality data in the United States. Jt Comm J Qual Patient Saf.

[35] Rozenblum R, Bates DW (2013). Patient-centred healthcare, social media and the internet: the perfect storm?. BMJ Qual Saf.

